# Functional modulation of the human gut microbiome by bacteria vehicled by cheese

**DOI:** 10.1128/aem.00180-25

**Published:** 2025-02-28

**Authors:** Christian Milani, Giulia Longhi, Giulia Alessandri, Federico Fontana, Martina Viglioli, Chiara Tarracchini, Leonardo Mancabelli, Gabriele Andrea Lugli, Silvia Petraro, Chiara Argentini, Rosaria Anzalone, Alice Viappiani, Elisa Carli, Federica Vacondio, Douwe van Sinderen, Francesca Turroni, Marco Mor, Marco Ventura

**Affiliations:** 1 Laboratory of Probiogenomics, Department of Chemistry, Life Sciences, and Environmental Sustainability, University of Parma 546768 , Parma, Italy; 2 Microbiome Research Hub, University of Parma 9370 , Parma, Italy; 3 GenProbio Srl 722140 , Parma, Italy; 4 Department of Food and Drug, University of Parma 9370 , Parma, Italy; 5 Department of Medicine and Surgery, University of Parma 478519 , Parma, Italy; 6 APC Microbiome Institute and School of Microbiology, Bioscience Institute, National University of Ireland 8796 , Cork, Ireland; The Pennsylvania State University, University Park, Pennsylvania, USA

**Keywords:** microbiota, food, metagenomics, metatranscriptomics, metabolomics, human diet

## Abstract

Since cheese is one of the most commonly and globally consumed fermented foods, scientific investigations in recent decades have focused on determining the impact of this dairy product on human health and well-being. However, the modulatory effect exerted by the autochthonous cheese microbial community on the taxonomic composition and associated functional potential of the gut microbiota of human is still far from being fully dissected or understood. Here, through the use of an *in vitro* human gut-simulating cultivation model in combination with multi-omics approaches, we have shown that minor rather than dominant bacterial players of the cheese microbiota are responsible for gut microbiota modulation of cheese consumers. These include taxa from the genera *Enterococcus*, *Bacillus*, *Clostridium,* and *Hafnia*. Indeed, they contribute to expand the functional potential of the intestinal microbial ecosystem by introducing genes responsible for the production of metabolites with relevant biological activity, including genes involved in the synthesis of vitamins, short-chain fatty acids, and amino acids. Furthermore, tracing of cheese microbiota-associated bacterial strains in fecal samples from cheese consumers provided evidence of horizontal transmission events, enabling the detection of particular bacterial strains transferred from cheese to humans. Moreover, transcriptomic and metabolomic analyses of a horizontally transmitted (cheese-to-consumer) bacterial strain, i.e., *Hafnia paralvei* T10, cultivated in a human gut environment-simulating medium, confirmed the concept that cheese-derived bacteria may expand the functional arsenal of the consumer’s gut microbiota. This highlights the functional and biologically relevant contributions of food microbes acquired through cheese consumption on the human health.

**IMPORTANCE:**

Diet is universally recognized as the primary factor influencing and modulating the human intestinal microbiota both taxonomically and functionally. In this context, cheese, being a fermented food with its own microbiota, serves not only as a source of nourishment for humans, but also as a source of nutrients for the consumer’s gut microbiota. Additionally, it may act as a vehicle for autochthonous food-associated microorganisms which undergo transfer from cheese to the consumer, potentially influencing host gut health. The current study highlights not only that cheese microbiota-associated bacteria can be traced in the human gut microbiota, but also that they may expand the functional repertoire of the human gut microbiota, with potentially significant implications for human health.

## INTRODUCTION

Scientific interest in the human gut microbiota has substantially expanded in recent years due to the growing number of studies showing a correlation between host health and the intestinal microbial ecosystem (1–3). Indeed, a multitude of studies have shown that the human gut microbiota is involved in various metabolic and physiological activities, including the degradation of otherwise non-digestible complex carbohydrates, education/modulation of the host immune system, protection against pathogen colonization, or provision of energy to intestinal epithelial cells to bolster intestinal barrier integrity. Moreover, it is also able to produce a plethora of bioactive compounds with a perceived positive impact on human health, for example, amino acids, vitamins, polyphenols, and/or short-chain fatty acids (SCFAs) (4–6).

Among the various factors affecting the composition and functionality of the gut microbiota, diet has been universally recognized as one of the main and consistently dominant drivers shaping this complex and intricate microbial ecosystem (7–15). Indeed, although diet acts primarily as a nutrient supply for humans and its associated microbiota, it also represents a potential transfer vehicle for food-associated microorganisms to the human gut, where they can proliferate, modulate gut microbiota composition, and influence host health (13, 16–19). In this context, especially fermented foods, which by definition are obtained “through desired microbial growth and enzymatic conversions of food components” (20), represent a particularly valuable dietary component. They may act as a source of nutritional, prebiotic, and bioactive compounds, selectively promoting growth and metabolic activity of particular intestinal bacterial species. Additionally, fermented foods have been proven to play a critical role in the horizontal transmission of diet-derived microorganisms, thereby increasing microbial biodiversity within the human gut, and ultimately influencing host health (21–23).

Among the many fermented foods, dairy products, in particular cheese and yogurt, are by far the most consumed worldwide (24–26). For this reason, the impact that these products can have on human health has been extensively studied, highlighting that these fermented foods possess unique features able to provide remarkable health benefits to the consumer (27, 28). For instance, the microbial activity occurring during dairy fermentations reduces the concentrations of high-calorie mono- and/or di-saccharides naturally present in milk, thereby reducing the glycemic index and enhancing food tolerability (16). Additional enzymatic transformations with crucial nutritional implications occur during milk fermentation, such as organic acid and amino acid production coupled with detoxification reactions and removal of anti-nutritive factors (29, 30). However, in addition to the modifications of the original food matrix which may indirectly and positively influence consumer health, bacterial fermentation of dairy products has also been shown to exert direct beneficial effects on the consumer (31). In this context, certain lactic acid bacteria that dominate the raw milk cheese microbiota may be transiently transferred the human gut, where they may exert positive effects, such as synthesizing bioactive compounds, inhibiting intestinal pathogens, and modulating epithelial function (32, 33). Furthermore, various investigations have shown the existence of a positive correlation between fermented dairy food consumption and weight maintenance (16, 34). Additionally, clinical trials have highlighted the potential beneficial roles of cheese consumption in enhancing the immune responses and alleviating the severity of rheumatoid arthritis symptoms by reducing inflammation and modifying the intestinal microbiota composition (28, 35–37).

However, although many studies have evaluated consumer health effects of cheese consumption, the impact of raw milk cheese dietary intake on the composition of the human intestinal microbiota or on its functional potential is still far from being fully understood.

Therefore, in the current study, we investigated the impact of cheese consumption on the taxonomic composition and functional potential of consumer gut microbiota, and on consumer health using an *in vitro* gut model combined with multi-omics approaches. Furthermore, to confirm that cheese consumption plays a role in modulating consumer gut microbiota, a strain-tracking analysis was performed to detect cheese microbiota-associated bacterial strains in fecal samples of cheese consumers.

## RESULTS AND DISCUSSION

### Investigation of the microbial community originating from *in batch* growth of raw milk cheeses in a human gut environment-simulating culture medium

An *in vitro* experiment was set up involving the *in batch* cultivation of 15 different raw milk-based cheeses in a gut environment-simulating medium (here referred to as GESM cultivation) for 16 h. In detail, this particular growth medium was selected not only because it mimics the variability and complexity of nutrients typical of the human intestinal environment, but also because it has been commonly exploited and validated in several studies to simulate the human colonic environment for *in vitro* cultivation (38–42). In addition to the medium composition, key physical and chemical parameters, including temperature, pH, and anaerobic conditions, were carefully checked to closely mimic the human gut environment, enabling an investigation into determining which bacteria from these raw milk cheeses are able to grow in the (simulated) human intestine.

**Fig 1:**
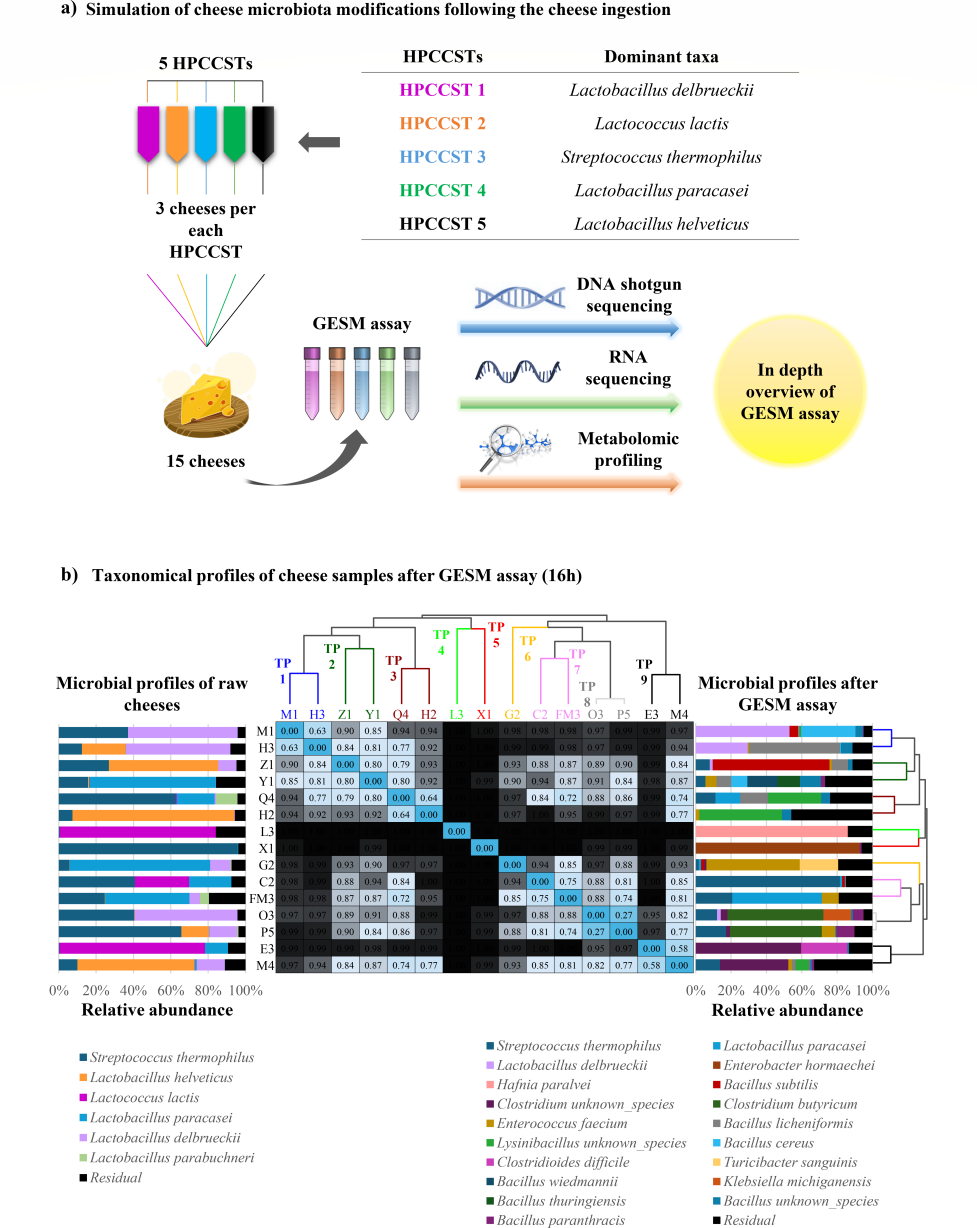
Microbial analysis workflow and taxonomic profile of cheese after cultivation in GESM. Panel a depicts the workflow followed to investigate the taxonomic, transcriptomic, and metabolomic outcomes obtained after a batch cultivation of raw milk cheese in a simulated gut environment. Panel b shows the hierarchical clustering approach based on Bray-Curtis matrix calculated on taxonomical profiles of cheese samples after GESM cultivation. In the center, the heat map displays the Bray-Curtis distance value matrix, highlighting the substantial taxonomic variability among cheese samples after GESM cultivation. Bar plots flanking the heat map illustrate the dominant bacterial species of raw cheese samples (left) and those cultivated on GESM medium (right). Only bacterial species with a maximum relative abundance of at least 10% across metagenomic samples were included.

The cheese samples were collected to broadly represent the most characteristic compositional patterns of Italian raw milk cheese microbiota. Indeed, the 15 cheese samples included three representatives of each of the five “High Prevalence Cheese Community State Types” (HPCCSTs), i.e., clusters of recurrent microbial profiles, previously identified in a study aimed at dissecting Italian raw milk cheese microbiota (Fig. 1a) (43). Both original cheese samples and bacterial cultures obtained after cheese GESM cultivation were subjected to DNA extraction and subsequent deep shotgun sequencing (Table S1), while the cultures from GESM cultivation were further investigated through RNA sequencing and metabolomic assessments (Fig. 1a). Analysis of the obtained taxonomic profiles revealed a drastic change in microbial composition following GESM cultivation when compared to the original cheese microbiota (Fig. 1b; Tables S2 and S3). Intriguingly, the most abundant and prevalent bacterial species of raw milk cheeses, i.e., *Streptococcus thermophilus* (average relative abundance of 29.7%), *Lactobacillus paracasei* (16.4%), *Lactobacillus delbrueckii* (15.2%), *Lactobacillus helveticus* (16.5%), and *Lactococcus lactis* (12.8%) (44), underwent a substantial decrease in their relative abundance when cheeses were cultivated in GESM (Fig. 1b; Tables S2 and S3). Indeed, only *Streptococcus thermophilus* maintained a high abundance (11.4%) and prevalence (60%) after GESM cultivation, while the other cited bacterial species showed an average relative abundance and prevalence that did not exceed 6% and 30%, respectively. In contrast, the relative abundance of various accessory bacterial taxa of the raw milk cheese microbiota showed a marked increase in GESM cultivation, including species belonging to the genera *Enterobacter*, *Bacillus*, *Clostridium,* and *Hafnia* (Fig. S3). Specifically, from 0.08% in L3 cheese, *Hafnia paralvei* relative abundance increased up to 86.07% in its corresponding GESM cultivation (Fig. S3a). Similarly, from a relative abundance not detectable in cheese samples, probably below the intrinsic detection limit of metagenomics methods, *Enterobacter hormaechei* and *Bacillus subtilis* reached a relative abundance of 92.72% and 66.07%, respectively, when X1 and Z1 cheeses were cultivated in GESM (Fig. 1b; Fig. S3a; Tables S2 and S3). These findings suggest that minor or accessory elements of the cheese microbiota are more likely to be responsible for potential taxonomic and functional modifications of the gut microbiota of the consumer, while cheese-dominant microbes produce metabolites with an important functional impact pertaining to the organoleptic features of dairy products, such as flavor, texture, or smell, along with metabolites affecting human health (45–47). Additional information concerning a detailed discussion on these taxonomic profiling results is reported in the supplemental text.

To further explore the microbial heterogeneity of the 15 assessed raw milk cheese samples following GESM cultivation, and to ultimately evaluate whether the obtained bacterial cultures may be still stratified into compositional patterns after growth in GESM, cheese cultured samples were clustered according to their microbial composition through a hierarchical clustering approach (HCA) (Fig. 1; Fig. S3b). The obtained results showed a high level of taxonomic diversity among samples following GESM cultivation, preventing the identification of distinct taxonomic clusters, as highlighted by the subdivision of the 15 samples into nine distinct taxonomic profiles (TPs) (Fig. 1b; Fig. S3b). This indicates that cheeses with an originally similar dominant taxonomic composition, following growth in an environment that mimics the human intestine, may result in different and variable outcomes in influencing the composition of the consumer gut microbiota. Additional details regarding the composition of the nine identified bacterial taxonomic profiles are reported in the supplemental text.

Overall, although the GESM cultivation model displays certain limitations, such as the absence of competing gut microbiota and stress conditions that cheese microbes encounter prior to reaching the colon (e.g., acid stress in the stomach and bile stress in the small intestine), these findings indicate that raw milk cheese consumption introduces a wide variety of bacterial species into the human gastrointestinal tract, including those that represent cheese microbiota sub-dominant taxa. However, if some cheese microbiota-associated bacterial species are able to transit or even (temporarily) persist in the gut of the consumer, they are expected to not only contribute to influence/expand the intestinal biodiversity, but also its metabolic potential. Therefore, an analysis of the transcribed genes as well as the produced metabolites is necessary to better understand how the cheese microbiota can impact consumer health.

### Assessment of the most highly transcribed enzyme-encoding genes by cheese-associated bacteria following GESM cultivation

To evaluate the functional contribution of cheese microbiota elements to the host gut microbial ecosystem when they reach and possibly (temporarily) colonize the human intestine, the 15 GESM cultivations were subjected to RNA extraction and sequencing to obtain associated metatranscriptome data sets (Fig. S3). These data sets were analyzed to identify transcripts related to enzyme-encoding genes to identify active metabolic pathways, and associated metabolites, of cheese-derived bacterial species when cultivated in a human gut-simulating environment (Table S4). Next, to determine whether the obtained transcriptional profiles could be stratified into distinct patterns, we performed hierarchical clustering, with the unsupervised Silhouette method identifying three optimal clusters, named expression clusters (EXCs). In detail, EXC1 and EXC3 were represented only by three samples, while the remaining nine samples fell into EXC2 (Fig. S1 and S3). This demonstrates that although GESM cultivation was shown to result in taxonomically diverse microbial assemblies, they nonetheless possess a high degree of consistency in enzyme-coding gene expression (analysis of similarity [ANOSIM] and permutational analysis of variance [PERMANOVA] *P*-value <0.001) (Fig. S1). These findings suggest that despite high taxonomic variability, the GESM communities may still exhibit similar expression profiles and may, therefore, express similar biological functions.

**Fig 2:**
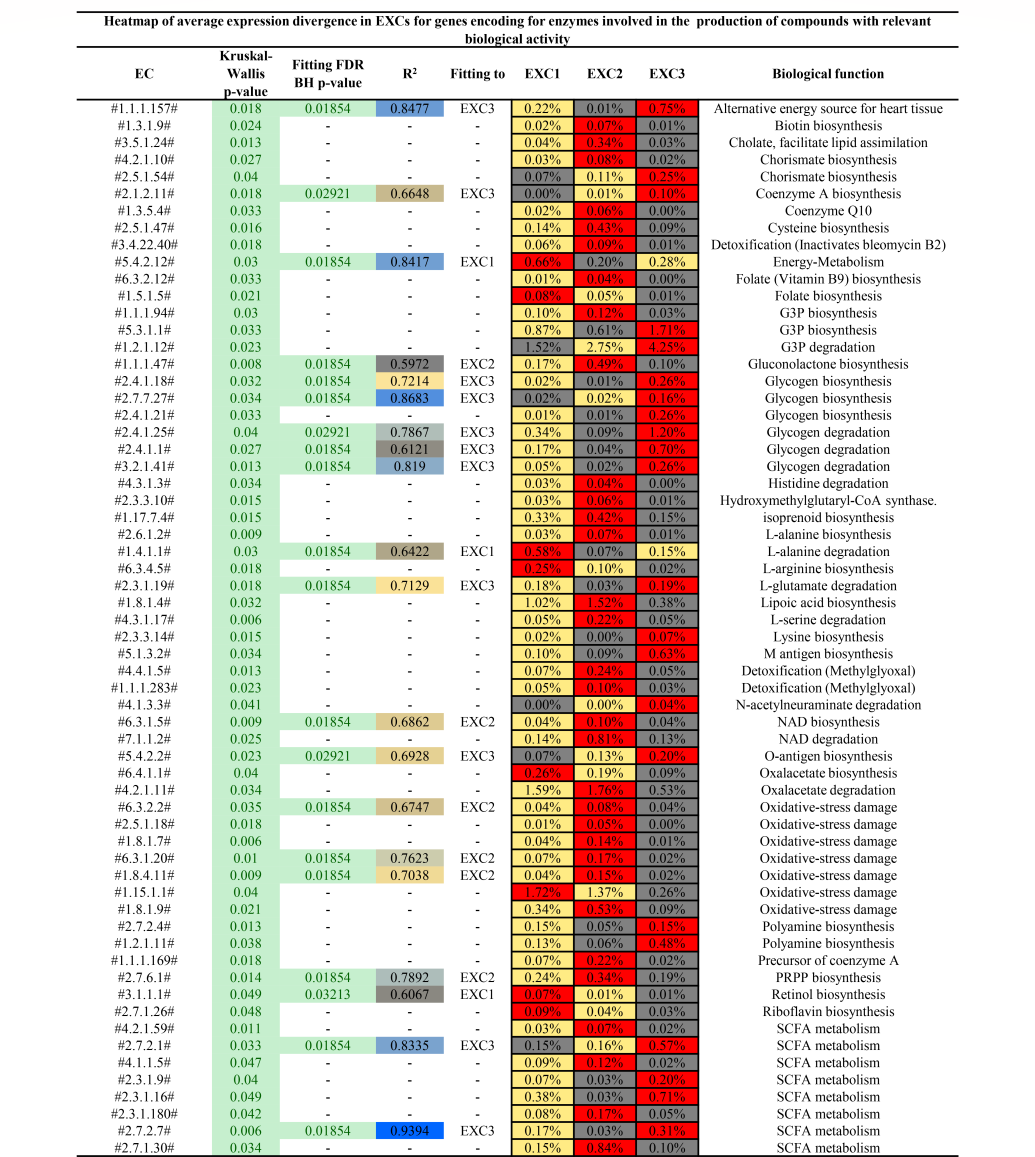
Differences in enzyme-coding gene expression levels among EXCs. The figure depicts an extract of all enzymatic functions with biological activity for which we identified statistically significant variances in the expression of their associated genes between the three EXCs. Kruskal-Wallis *P*-value column explains the significance obtained through Kruskal-Wallis statistical analysis, with the green-highlighted cells containing statistically significant values (*P*-value <0.05). The fitting FDR with Benjamini-Hochberg correction *P*-value column shows the statistical significance of the fitting analysis, which identified enzymatic functions (classified by their EC number) that best explain gene expression biodiversity in the studied data. Instead, the variability explained by the EC numbers found to be significant from the fitting analysis is shown in column *R*^2^, with values that range from lowest (gray) to highest (blue). The EXC columns depict the average gene expression values relative to the selected EC numbers, with the maximum values in red and the lowest values in gray.

To evaluate if the three identified EXCs provide a different functional contribution to the intestinal microbiota of the consumer, a fitting analysis was performed (false discovery rate [FDR]-corrected Benjamini-Hochberg [BH] empirical *P*-value <0.05 and *R*^2^ >0.5) along with a non-parametric Kruskal-Wallis test (*P*-value <0.05) (Fig. S1a; Table S5). In addition, the MetaCyc database (25) was used to manually assign a function to each enzyme-coding gene with a statistically supported different average expression in the EXCs. This analysis identified 62 enzyme-coding genes (ECGs) (functionally classified by their Enzyme Commission [EC] number), whose transcription levels were shown to be significantly different among the three EXCs. Interestingly, all these genes encode enzymes responsible for the production of compounds which may have direct or indirect implications for human host physiology if released in the gut lumen by active transport or as a result of bacterial cell lysis (Fig. 2; Table S5). Among the enzymatic reactions corresponding to differentially transcribed genes, ECGs implied in the biosynthesis of SCFAs (48), as well as in the biosynthesis of coenzymes (Q10 and A) (49), vitamins (biotin, folate, and riboflavin) (50), and protection against oxidative stress (glutathione biosynthesis) were identified. In addition, transcription levels of certain genes encoding enzymes involved in metabolic pathways dedicated to detoxification of dangerous molecules or to amino acid biosynthesis (i.e., L-glutamate and L-arginine) (51) were significantly different among the three EXCs (Fig. 2; Table S5). Notably, genes involved in vitamin and amino acid production showed significantly higher transcript levels in all EXCs, suggesting that cheese microbiota-associated bacterial species that reach the intestine are indiscriminately able to produce these biologically relevant compounds that positively influence, if released, both the intestinal microbiota and the host itself. In contrast, other enzymatic functions seemed to be specific for one or two EXCs. Specifically, a significant number of transcripts related to genes involved in preventing oxidative stress damage and SCFA production was recorded for EXC2 and EXC3 compared to EXC1, while transcription of genes involved in bile acid detoxification was significantly higher in EXC2 only (Fig. 2; Table S5). Therefore, as expected, the cheese microbiota may have a significant impact on the intestinal microbiota and ultimately on consumer health as based on the identified transcribed genes associated with predicted enzymatic pathways involved in the production of beneficial molecules. However, the gene expression response is not univocal when cheeses are grown in GESM but rather constitutes significant differences that probably depend on the bacterial species that are able to reach/proliferate in this environment.

In this context, to determine which bacterial species of the GESM cultivations possessed the 62 genes that were differentially transcribed among the three EXCs, a gene back-tracking analysis was performed (Fig. S1b). Interestingly, although this analysis highlighted the presence of these genes in some of the dominant species of the cheese microbiota, including *Lactobacillus delbrueckii*, *Lactobacillus paracasei*, and *Streptococcus thermophilus*, the highest abundance of these genetic sequences was detected in certain bacterial taxa that were enriched after GESM cultivation, i.e., *Enterobacter hormaechei*, *Hafnia paralvei*, and *Clostridium butyricum* (Fig. S1b). This suggests that these less abundant members of the cheese microbiota may have a significant impact on human health when they reach the intestine after cheese consumption.

Overall, these results support the notion that although differences in terms of transcriptional profiles are observed, cheese microbiota-associated bacterial species play an important role in enhancing or expanding the functional potential of the intestinal microbiota of this dairy product consumer. Based on this intriguing outcome, to further investigate the metabolic repertoire of these microbial populations and their abilities to shape the metabolome of the growth environment after GESM cultivation, we performed a metabolomic analysis.

### Metabolomic investigation of bacterial compounds produced after GESM cultivation

To evaluate the metabolic modulation induced by the cheese-associated microbiota following GESM cultivation, an untargeted metabolomic investigation was performed using liquid chromatography coupled with high-resolution mass spectrometry (LC-HRMS) on the 15 GESM cultivations. The untargeted metabolomic strategy enabled the detection of 2,411 LC-HRMS features, which were subsequently refined to 1,787 based on a log_2_ difference greater than 1 or less than −1 in signal intensities relative to the incubation medium in at least 1 of 15 GESM assay samples (Table S6).

**Fig 3:**
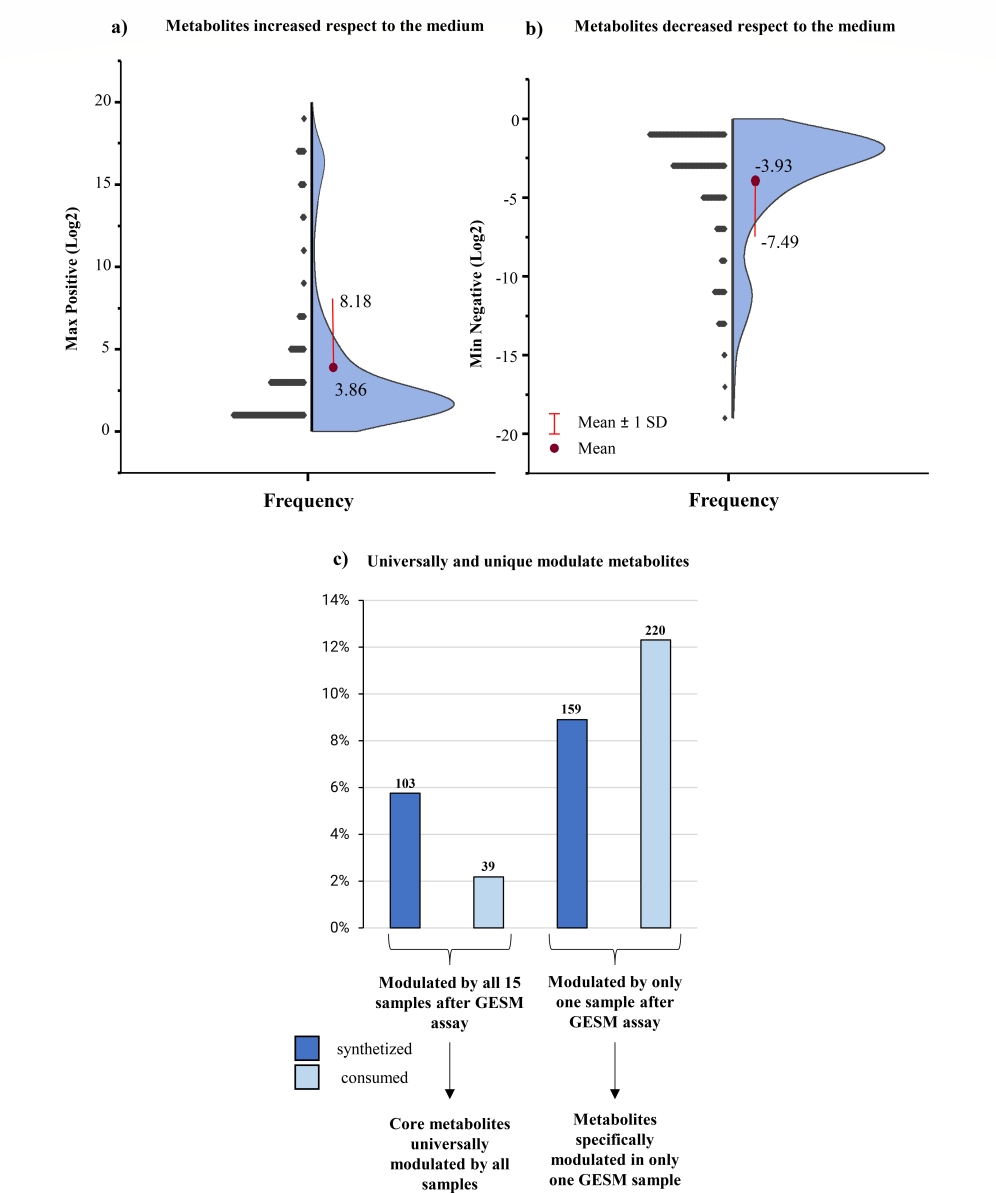
Metabolomic analysis of GESM cultivations. Panels a and b show the violin plot of log_2_ distribution profiles of metabolites with significant signal following cheese microbiota growth in GESM (log_2_ >1 or log_2_ <−1) with respect to the medium without inoculum. Panel c depicts the bar plot of metabolite signals increased (or appeared *de novo*) and decreased in all GESM cultivation samples compared to those modified in a single GESM cultivation sample.

The analysis of signal intensities revealed that among the 1,787 selected LC-HRMS signals, 103 either increased or appeared *de novo*, while 39 were decreased in all GESM cultivations when compared to the control, which consisted of the gut-simulating medium with or without the addition of the heat-treated cheese (Fig. 3; Table S6). These 142 signals can therefore be regarded as a set of “core metabolites” that consistently exhibit a universal and unitary change in abundance during the 16 h of incubation of cheese samples in GESM. In detail, 26 out of the 103 increased signals and 19 out of the 39 decreased signals had a putative ID assigned by comparison of the detected HRMS features (accurate mass, fragmentation profile, isotopic similarity) with those available in public databases (HMDB, KEGG, ChEBI). These compounds represented a pool of metabolites (amino acids, small peptides, phenolic compounds, acylcarnitine, fatty aldehydes and acids, sterols, and phosphoglycerolipids) originating from the cheese microbiota-mediated enzymatic biotransformation of compounds derived from either the gut-simulating incubation medium or cheese matrix used for inoculum.

Following metabolite identification, and in order to evaluate if different transcriptional profiles (EXCs) also correspond to different metabolic patterns, a correlation analysis was performed between metabolite intensities and the three identified EXCs. This analysis highlighted a set of 90 metabolites whose abundance was significantly different among the three EXCs (Kruskal-Wallis *P*-value <0.05) (Table S7). Notably, 26 possessed a putative compound identity (ID) based on available reference databases (Table 1; Table S7).

**TABLE 1:** Metabolites with predicted ID showing significant differences among the three identified EXCs

Met ID	Class	Predicted compound name	GESM chemical formula	RT (min)	*m/z*	Mass error (ppm)	EXC1	EXC2	EXC3
Met124	Amino acids	S-(α-(Hydroxyimino)-4-hydroxyphenyl)-L-cysteine	C11H14N2O4S	14.6	253.0634	−2.62	−3.13	0.84	−2.97
Met1081	Amino acids	DL-phenylalanine	C9H11NO2	12.11	148.0755	−0.86	0.08	0.04	0.85
Met184	Amino acids	N-myristoylglycine	C16H31NO3	20.18	286.2377	0.02	−3.35	−0.37	−1.33
Met2405	Dipeptides	Glu-Glu	C10H16N2O7	6.35	259.0926	0.5	−1.41	−2.43	−2.4
Met2043	Dipeptides	Gamma-glutamylvaline	C10H18N2O5	5.26	247.1289	0.13	−1.91	−2.88	−3.56
Met947	Oligopeptides	Pepsinostreptin	C33H61N5O9	11.59	710.4078	−3.32	0.14	1.4	1.3
Met470	Oligopeptides	Leu-Gly-Pro	C13H23N3O4	10.5	268.1656	0.13	−0.31	0.34	1.12
Met243	Oligopeptides	Tetragastrin	C29H36N6O6S	19.28	597.2515	4.22	−1.54	−1.62	−4.69
Met681	Glycerolipids	3-Oxohexadecanoic acid glyceride	C19H36O5	19.89	367.2450	−1.4	3.73	1.92	3.97
Met1052	Glycerophospholipids	1-Palmitoylglycerone 3-phosphate	C19H37O7P	8.36	431.2160	−2.19	2.47	1.62	3.68
Met107	Acylcarnitines	Hydroxyhexanoylcarnitine	C13H25NO5	11.71	298.1638	4.65	7.76	11.1	11.96
Met2157	Fatty acyl glycosides	DHZ-9-NGOG	C22H35N5O11	10.03	528.2304	0.66	−0.32	−0.99	-1
Met669	Fatty aldehydes	(8E)-8-Heptadecenal	C17H32O	20.72	253.25261	0.08	6.92	10.62	8.59
Met351	Fatty aldehydes	2,4-Hexadienal	C6H8O	9.21	79.0541	−1.67	3.59	4.74	4.52
Met646	Fatty acids	4-Hydroxy-2-oxobutanoic acid	C4H6O4	4.82	101.0232	−0.92	1.95	1.1	1.5
Met572	Fatty acids	–^*a*^	C12H24O2	20.82	183.1744	0.28	3.74	2.1	5.04
Met432	Fatty acids	–	C18H32O4	20.04	295.2267	−0.4	−0.94	−0.22	−0.83
Met498	Fatty acids	–	C18H36O4	20.72	299.2581	0.22	7.58	9.42	7.03
Met149	Fatty acids	Octadecenedioic acid	C18H32O4	19.04	295.2257	−3.44	−0.72	−0.35	−1.09
Met2180	Fatty acids	–	C18H32O2	19.53	263.2369	−0.01	0.86	1.96	1.15
Met684	Fatty acids	–	C18H34O3	19.53	298.2509	0.36	1.8	3	1.95
Met1343	Sterol lipids	Glycocholic acid	C26H43NO6	15.82	466.3163	0.01	−1.77	−3.38	−7.44
Met502	Sterol lipids	Taurodeoxycholic acid	C26H45NO6S	19.28	500.3040	−0.04	−1.43	−1.74	−4.93
Met101	Phenols	2,3-Xylenol	C8H10O	9.2	105.0698	−0.66	7.01	8.23	7.93
Met23	Purine metabolites	5-Aminoimidazole-4-carboxylic acid	C4H5N3O2	4.03	110.0348	−0.48	6.99	6.07	7.1
Met112	Secondary metabolites	5-Me-2-Fur-THP	C10H13NO	9.21	146.0964	−0.42	9.88	11.28	10.94

^*a*^ Dashes indicate metabolites for which no identity has been assigned.

Among the latter, the relative abundance of various amino acids, dipeptides, and oligopeptides significantly decreased in EXC1 or EXC3, suggesting a high proteolytic activity index due to protein degradation for these two EXCs (Table 1). At the same time, GESM cultivations belonging to the EXC2 were characterized by a significantly higher relative abundance of a phenolic compound, two fatty aldehydes, and five out of the seven identified metabolites corresponding to fatty acids (Table 1; Table S7). These findings suggest that EXC2 represents the most metabolically prolific transcriptional profile.

Furthermore, although exhibiting a significant lower average relative abundance in EXC3 compared to the other two EXCs, a remarkable reduction in two sterol lipids corresponding to bile acids was observed across all three EXCs when compared to the control sample, i.e., the GESM alone (Table 1; Table S7). This observation suggests the ability of certain cheese microbiota-associated bacterial species to metabolize bile acids. The latter are generally present in the intestine as primary bile acids conjugated to either taurine or glycine. However, certain bacterial players of the gut microbiota can deconjugate primary bile acids to release taurine, glycine, and secondary bile acids. The latter are widely known to protect against the invasion and proliferation of certain opportunistic pathogens, regulate the gut microbiota composition through their antimicrobial activity, and/or modulate intestinal homeostasis and immunity (52–54). Therefore, it can be argued that certain cheese microbiota-associated bacterial species, when reaching the cheese consumer intestine, play a role in influencing host health through bile acid metabolism.

However, despite significant differences in the relative abundance of the produced metabolites among EXCs, metabolite alterations (increase or reduction) were consistent among the three EXCs when compared to the control. This suggests that different EXCs are able to produce similar metabolites, yet at different levels, while it also supports the notion that different cheese taxonomic profiles converge into similar metabolic functions.

Therefore, although the *in vitro* model used does not precisely mimic the different environments to which food is exposed during the transit through the whole human gastrointestinal tract prior reaching the large intestine, the obtained results support the notion that consuming fermented foods, such as cheese, may affect the host gut metabolome, possibly impacting on host well-being. This effect is thought to be caused by the complex rearrangement of metabolites as a result of the action of microbiota metabolism within the human gut that occurs following cheese ingestion, as simulated here by means of GESM cultivation. Therefore, an intriguing question arises regarding the integration of biologically relevant microbial genetic features and the resulting metabolomic shuffling into the human gut microbiome as a result of raw milk cheese consumption.

### Validation of cheese as a contributor of cheese microbiota-associated metabolic functions to the gut of the consumer

**Fig 4:**
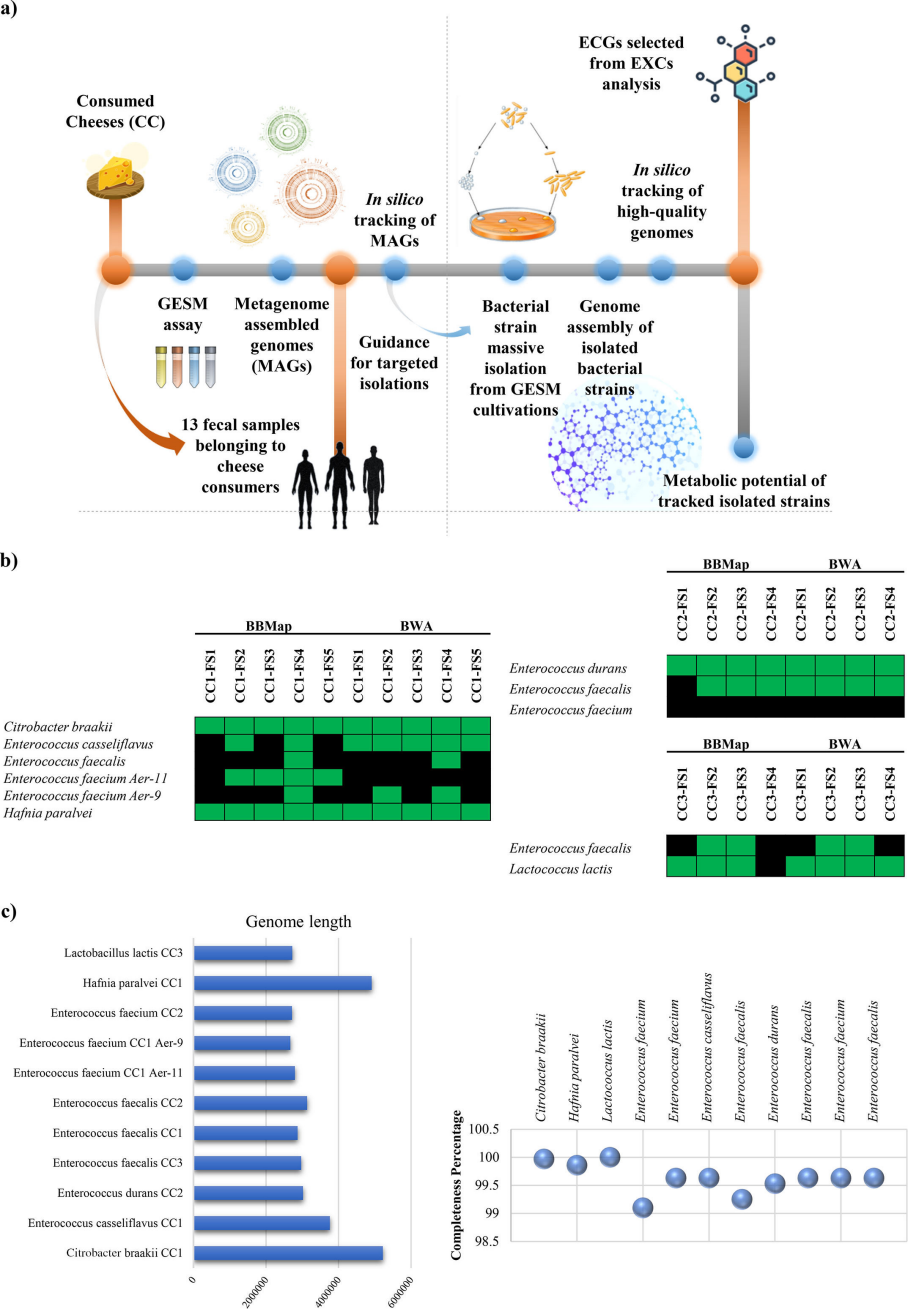
Multi-omics evaluation of cheese-to-consumer horizontal transmission of bacterial strains. Panel a illustrates the workflow followed to validate transmission of metabolic functions from the microbiota present in cheeses to the gut microbiome of consumers through metagenomics, culturomics, genomics, and transcriptomics approaches. Panel b shows a series of heat maps related to metagenomic strain tracking performed using BBMap and BWA methods, with green cells indicating positive hits with more than 10,000 covered bases. Panel c provides the completeness level scores of the isolated strains and their total genome length based on CheckM analysis.

In order to validate the hypothesis that cheese microbiota plays a role in modulating/expanding the functional potential of human intestinal microbiota following ingestion of dairy products, the presence of cheese-associated bacterial strains in the gut microbiota of cheese consumers was investigated. For this purpose, three raw milk cheese samples (consumed cheese, CC) named CC1, CC2, and CC3 as well as 13 fecal samples belonging to healthy consumers of these three cheeses were collected. Specifically, five individuals consumed CC1, while four individuals consumed CC2 and an additional four consumed CC3. To evaluate which bacterial species of the cheese microbiota may modulate composition and/or functional potential of the gut microbiota of cheese consumers, the three CC were cultivated in GESM for 16 h. Subsequently, fecal samples as well as the original cheese samples and the corresponding GESM cultivations were subjected to bacterial DNA extraction and deep shotgun sequencing (Table S8). Interestingly, also for these three cheeses, as already observed above, the cultivation of the fermented foods in a cultivation medium that simulates the human gut environment induced a preponderant growth of minority bacterial species of the cheese microbiota, confirming the trend observed above (Table S8). Indeed, while CC2 and CC3 were dominated by *Streptococcus thermophilus* and CC1 by *Leuconostoc mesenteroides*, i.e., two bacterial species typical of the cheese microbiota (55–57), their corresponding GESM cultivations were characterized by a predominance of species belonging to the genus *Clostridium* (CC2 and CC3 GESM cultivation) or *Hafnia paralvei* (CC1 GESM cultivation) (Table S8). Based on the sequencing data, to assist in the isolation of bacterial strains that may have been transferred from cheese products to the gut of a human consumer, an *in silico* strain-tracking approach, based on metagenome-assembled genomes (MAGs), was employed (Fig. 4a). Specifically, a total of 57 MAGs and contigs with a minimum length of 10,000 bases were reconstructed from microbial shotgun metagenomics data of the three cheese samples following GESM cultivation (Table S9). Next, in order to trace the identified GESM cultivation-associated MAGs in the fecal samples of the 13 cheese consumers, the reconstructed MAGs were used to guide a preliminary *in silico* strain-tracking effort by scrutinizing shotgun metagenomics data obtained from the fecal samples of the 13 enrolled consumers. This analysis allowed detection and tracking of 15 different microbial genera and 36 bacterial species putatively transferred from the cheese product to the human gut (Table S10). These *in silico* data of putative cheese-to-consumer transferred bacteria were exploited to guide a matrix-assisted laser desorption/ionization time-of-flight mass spectrometry (MALDI-TOF) Biotyper-based large-scale/high-throughput isolation effort aimed at recovering those strains from GESM cultivation samples. This approach allowed the isolation of 11 strains corresponding to seven bacterial species, i.e., *Citrobacter braakii*, *Hafnia paralvei*, *Lactococcus lactis*, *Enterococcus faecium*, *Enterococcus faecalis*, *Enterococcus durans*, and *Enterococcus casseliflavus*. As expected, due to the inherent challenges of cultivating intestinal bacteria, not all detected species were successfully cultivated under laboratory conditions. In this regard, the selection process was dependent on the culturability of the strains, rather than on their abundance in the fecal samples, reflecting a common limitation in microbiological studies. Subsequently, to evaluate the functional potential of these isolates, each bacterial strain was subjected to sequencing and genome reconstruction through MEGAnnotator2 software (Table 2; Fig. 4a) (58).

**TABLE 2:** Strains isolated from the GESM cultivation of CC samples and general genome features of the 11 newly isolated bacterial strains

Genome	Isolation	Completeness (based on CheckM)	Coverage	Bacterial species	ANI vs indicated species
Genome 1	CC1-GESM	99.97	×98	*Citrobacter braakii*	93.4
Genome 2	CC1-GESM	99.86	×101	*Hafnia paralvei*	99
Genome 3	CC3-GESM	100	×131	*Lactococcus lactis*	98.07
Genome 4	CC3-GESM	99.1	×65	*Enterococcus faecium*	94.77
Genome 5	CC1-GESM	99.63	×171	*Enterococcus faecium*	94.82
Genome 6	CC1-GESM	99.63	×151	*Enterococcus casseliflavus*	97.97
Genome 7	CC1-GESM	99.25	×165	*Enterococcus faecalis*	98.81
Genome 8	CC1-GESM	99.53	×169	*Enterococcus durans*	99.41
Genome 9	CC2-GESM	99.63	×187	*Enterococcus faecalis*	98.61
Genome 10	CC2-GESM	99.63	×118	*Enterococcus faecium*	94.84
Genome 11	CC2-GESM	99.63	×158	*Enterococcus faecalis*	98.89

Intriguingly, in-depth insights into the reconstructed genomes revealed that the bacterial isolates possessed several genes in their genetic arsenal predicted to encode various metabolic functions that match the metabolome of the cheese microbiota grown in the GESM environment as reported above (Table S11). In detail, among the bacterial isolates, *Hafnia paralvei* strain T10, isolated from CC1-associated GESM cultivation, was shown to possess a genome with the highest enzymatic potential encompassing 52 out of the 62 ECGs identified above (Table S12). These findings suggest that the (transient) presence of *H. paralvei* in the intestine of a cheese consumer significantly contributes to the functional potential of the intestinal microbiota and possibly produces various metabolites that may influence not only the intestinal microbiota, but also consumer health. Consistent with this notion, previous studies reported that members of the genus *Hafnia* possess a unique functional potential, responsible for the production of a diverse set of metabolites, including vitamins, SCFAs, and bacteriocins. These metabolites not only contribute to the organoleptic properties of cheeses but may also exert beneficial effects on the human host (59, 60).

To validate apparent transmission of the isolated strains, the availability of high-quality genomes reconstructed from chromosomal DNA sequencing was employed for additional and more accurate tracking approaches (Fig. 4b). In detail, two different mapping methods (based on BBMap and BWA software, detailed in Materials and Methods) were used to confirm transmission of these 11 isolated strains into the human gut (Fig. 4b and c). The results obtained highlighted the presence of *Enterococcus durans* (isolated from GESM CC2), *Citrobacter braakii* (isolated from GESM CC1), and *Hafnia paralvei* (isolated from GESM CC1) in all fecal samples of consumers of the corresponding raw milk cheese (Fig. 4b). The latter corroborates our presumption that cheese microbiota-associated bacterial species can reach the human intestine and possibly modulate the composition/functionality of the intestinal microbiota, potentially influencing consumer health.

### *Hafnia paralvei* as a model to investigate transmission of relevant microbial genetic features from raw cheese to the consumer human gut

To further confirm that cheese consumption can promote horizontal transmission of cheese microbiota-associated bacterial species to the gut microbiota of the consumer, favoring the expansion of the functional potential of the intestinal microbiota itself, *Hafnia paralvei* strain T10 was further explored as a model for further multi-omics validations. Specifically, *H. paralvei* T10 was selected as model strain since it was one of the 11 bacterial isolates from CC samples containing the highest number of ECGs (52 out of the 62 identified ECGs) (Fig. S1b; Table S11).

Metagenomic data derived from the sequencing of cheese consumer fecal samples revealed the presence of *H. paralvei* only in one out of the five fecal samples belonging to CC1 consumers (Table S8). However, metagenomic methods have intrinsic detection limits whereby a bacterial species below a certain relative abundance may not be detected. For this reason, to first validate the presence of this strain in the gut microbiota of the consumers, a strain-specific primer pair was designed to amplify a *Hafnia paralvei* T10 strain unique genomic trait based on the high-quality genome sequence obtained for this strain. DNA extracted from fecal samples belonging to CC1 consumers was then subjected to quantitative real-time PCR (qRT-PCR). Interestingly, even if with a very low abundance for certain samples, as expected for a non-autochthonous bacterial strain of the human intestine, the strain was detected in each analyzed fecal sample belonging to CC1 consumers (Table S12), indicative of the presence of *Hafnia paralvei* strain T10 in the gut of raw milk cheese consumers.

To evaluate if the ECGs present in the *H. paralvei* T10 genome are actively transcribed when the microorganism is in an intestinal environment, the strain was cultivated in GESM with and without heat-treated cheese. Specifically, this second condition was selected to evaluate if the presence of the cheese matrix plays a role in inducing transcription of ECGs in the strain under consideration. Following growth, cells were harvested and subjected to RNA sequencing. Remarkably, RNAseq analyses showed that *H. paralvei* T10 was able to actively express 45 ECGs, regardless of the culture condition (Table S13), as indicated by an RPKM (reads per kilobase of transcript per million mapped reads) >10, a robust threshold selected to avoid false positives (61). Indeed, only four ECGs were expressed with an RPKM >10 in only one of the two considered conditions, indicating that ECGs are actively transcribed by *H. paralvei* T10 when grown in GESM regardless of the presence of the cheese matrix. In detail, *H. paralvei* T10 showed transcription of genes involved in the increase of the overall antioxidant capacity of the gut environment (e.g., gene encoding for sulfur-containing protein like glutathione [GSH] or involved in lipoate biosynthesis) (Table S13) (62–64). Additionally, genes related to amino acid biosynthesis (e.g, L-cysteine and L-arginine) and SCFA metabolism were shown to be highly transcribed (Table S13).

To further confirm the role played by this strain in the human gut, metabolomic analyses were conducted on the supernatant recovered from *H. paralvei* T10 grown in GESM or GESM supplemented with heat-treated cheese. The obtained data, analyzed using partial least squares discriminant analysis (PLS-DA), revealed significant changes in the levels of various compounds. These changes, expressed as log2 differences, were observed under both growth conditions when compared to the sterile cultivation medium (Fig. S2; Table S14). These mainly included representatives of glycerophospholipids and oligopeptide derivatives (Fig. S2; Table S14).

Intriguingly, *H. paralvei* T10 grown on GESM and GESM with heat-treated cheese were shown to exhibit a high level of metabolic activity, which influenced the composition of the surrounding environment through the release of bacteria-derived compounds. These findings, therefore, provide compelling evidence for the notion that bacteria harbored by raw milk cheese can effectively expand the human gut metabolome.

### Conclusions

The combination of *in vitro* experiments using a human gut environment-simulating culture medium and omics-based analyses allowed us to demonstrate that low-abundance cheese microbiota-associated bacterial species, rather than their high-abundance counterparts, are the most effective food bacteria influencing the taxonomic composition and associated functional potential of consumer gut microbiota. Although this model undoubtedly lacks the natural complexity of the gut environment, such as exposure to the human intestinal mucosa and host-produced compounds and interaction with the native bacterial community, it represents a valuable exploratory tool, thus providing intriguing preliminary data that will guide future research. Results obtained through this approach revealed that this taxonomic heterogeneity may result in similar expression profiles, thereby suggesting the potential for comparable functional repertoires. Specifically, cheese microbiota-associated bacterial species that successfully proliferated in our human gut environment-simulating model appear to influence gut microbiota composition and host health. This is achieved through active expression of genes implied in the production of various beneficial metabolites, ultimately contributing to the expansion of the human gut microbiota functional potential.

In this context, to further validate the hypothesis of horizontal transfer of cheese-associated bacterial strains and their metabolic functions to the human gut, a strain-tracking approach was conducted involving habitual cheese consumers. This analysis identified several cheese-derived species capable of reaching the gut of these individuals. In this context, *Hafnia paralvei* T10 was selected as a model strain to demonstrate the actual transmission of bacteria from cheese to the consumer. Furthermore, the presence of this strain in the gut was shown to contribute to the synthesis of a range of metabolites with potential roles in microbe-microbe and microbe-host interactions. However, further *in vitro* experiments are needed to evaluate the viability of cheese microbiota-associated bacterial strains upon reaching the consumer’s gut as well as their interactions with the native gut microbiota. Overall, these findings demonstrate the relevance of fermented foods, such as raw milk cheese, in contributing to the complexity, diversity, and activity of the gut microbiome, with a potential positive impact on human health and well-being.

## MATERIALS AND METHODS

### Cheese sample collection

Three cheese samples, each from a different type of raw milk cheese, were collected per each of the five recently described HPCCSTs (43), resulting in a total of 15 Italian cheese samples. As raw milk-derived cheeses, the collected samples were not subjected to pasteurization or other heat treatments. However, since cheese production methods can vary from producer to producer, no precise information regarding production temperature or acidification level was available for the collected samples. Almost 200 g of each cheese product was kept on ice and shipped to the laboratory under frozen conditions and vacuum packed. Once in the laboratory, cheese samples were immediately processed. To reduce the risk of environmental contamination, cheese samples were collected from an internal region of the whole cheese wheel and were further processed under sterile conditions.

### Cheese microbiota growth in a human gut-simulating medium through *in batch* cultivation

To evaluate which bacteria among the cheese microbiota are capable of proliferation in the human gut following cheese consumption, the 15 collected cheeses were cultivated as a batch culture in a previously described human gut environment-simulating growth medium (38, 65–67). This growth medium closely resembles the human intestinal environment by adding key components, such as bile salts, and mucin, while also strictly regulating critical parameters including temperature, pH, and anaerobiosis (38, 43, 67).

Specifically, cultivations were performed in triplicate by inoculating 0.1% (wt/vol) of a specific cheese in 30 mL of GESM. Inoculated cheese portions were collected under sterile conditions to minimize potential contamination from the surrounding environment. All cultures were incubated under anaerobic conditions at 37°C. After 16 h of incubation, all cultures showed a total bacterial count ranging from 8.8 × 10^6^ to 6.6 × 10^8^, as determined by flow cytometry. Since our intention was to highlight overall shifts in dominant taxa rather than explore minor changes at the individual species level, a single random culture for each of the 15 cheeses was employed for DNA sequencing. Notably, each HPCCST was still represented by three distinct samples, covering the natural inter-cheese microbiota micro-diversity. Thus, 5 mL was collected for DNA extraction and stored at −20°C, while the remaining volume was centrifuged at 5,000 × *g* for 5 min. The obtained pellets were then used for RNA extraction, while the supernatants were employed for metabolomic analyses. Both pellets and supernatants were stored at −80°C until they were processed.

### DNA extraction and sequencing

For extraction of DNA from cheese samples, in an effort to avoid inclusion of the rind, a fixed amount of 1 g of raw milk cheese belonging to the central portion was homogenized with 9 mL of phosphate-buffered saline (PBS; pH 6.5). Subsequently, 1.5 mL of each resuspended cheese sample was subjected to bacterial DNA extraction using the DNeasy PowerFood Microbial Kit (Qiagen, Germany), according to the manufacturer’s instructions. Subsequently, the concentration and purity of the extracted DNA was evaluated by using a spectrophotometer (Eppendorf, Germany). The extracted DNA was prepared using the Illumina Nextera XT DNA library preparation kit. Briefly, the DNA samples were enzymatically fragmented, barcoded, and purified involving the Agencourt AMPure XP DNA purification magnetic beads (Beckman Coulter Genomics GmbH, Bernried, Germany). Samples were then quantified using the fluorometric Qubit quantification system (Life Technologies, USA), loaded on a 2200 TapeStation instrument (Agilent Technologies, USA), and normalized to 4 nM. Sequencing was performed using an Illumina NextSeq 500 sequencer with NextSeq high output v2 kit chemicals (150 cycles) (Illumina Inc., San Diego, CA 92122, USA).

For DNA extraction from cultures obtained after cheese growth in GESM, 200 µL of each batch fermentation was subjected to DNA extraction using the QIAmp Fast DNA Stool Mini Kit (Qiagen, Germany) following the manufacturer’s instructions. The extracted DNA was then prepared as above described for DNA extracted from cheese samples.

### Metagenomics data processing, taxonomic profiling, and functional analysis

Each metagenomic data set was filtered to remove reads with a quality of <25 (score obtained from FastQC software for Illumina sequencing) and reads from *Bos taurus*, while reads with a length of >149 bp were retained. Quality-filtered data were then used for taxonomic and functional profiling through the METAnnotatorX2 bioinformatics platform, as previously described (68, 69). Megablast was employed for the taxonomic classification of each metagenomic read, using a curated non-redundant sequence database of genomes retrieved from NCBI servers and manually selected. The generation of the taxonomical database was reported in detail by Milani et al. (68) and periodically updated (every 6 months). Reads with a nucleotide identity of >94% to reference genomes were classified at the species level, while reads with a lower percentage identity were classified at the genus level as undefined species.

In addition, the functional enzymatic classification of each metagenomic read was performed through DIAMOND, employing a curated non-redundant sequence database of EC number sequence created employing the MetaCyc database. The default parameters set in the METAnnotatorX2 pipeline were used for this analysis using up to 5,000,000 reads (--query-cover 80, -evalue 0.00000001, and --max-target-seqs 1). Taxonomic EC back-tracking analysis was performed using the “-x ec_taxonomy” function of the METAnnotatorX2 pipeline, allowing the identification of those bacterial species possessing, in their genomes, the genetic sequences corresponding to a selected Enzymatic Code list.

For those analyses that required the use of the R software, version R-4.1.2 was used, along with the version RStudio2021.09.2-382 for R Studios and rtools40v2-x86_64 for rtools.

Similarities between samples (beta-diversity) were calculated using the Bray-Curtis distance matrix based on species relative abundance with the vegdist function (from vegan_2.5-7) on R-Studios (2020, RStudio: Integrated Development for R. RStudio, PBC, Boston, MA). The range of similarities is calculated between values 0 and 1. Beta-diversity was represented through principal coordinate analysis (PCoA) using ORIGIN 2023 (https://www.originlab.com/2023).

In the PCoA, each dot represented a sample, distributed in tridimensional space according to its bacterial composition, i.e., eigenvalue scores. The HCA of samples, performed on ORIGIN 2023, was achieved employing Bray-Curtis matrix using Pearson correlation as a distance metric and the sum square of distances and furthest neighbor for clustering methods. A Silhouette analysis performed on ORIGIN 2023 was employed to define the optimal number of clusters.

### RNA extraction and sequencing

Total RNA from each GESM-based cheese culture was isolated using a previously described method (70). Briefly, bacterial cell pellets were resuspended in 1 mL of QIAzol lysis reagent (Qiagen, Germany) in a sterile tube containing glass beads (Merck, Germany). Cells were lysed by alternating 2 min of stirring the mix on a bead beater with 2 min of static cooling on ice. These steps were repeated three times. The lysed cells were centrifuged at 13,000 × *g* for 15 min, and the upper phase was recovered. RNA was then purified using the RNeasy Mini Kit (Qiagen, Germany) following the manufacturer’s instruction. RNA concentration and purity were evaluated using a spectrophotometer (Eppendorf, Germany). For RNA sequencing, total RNA (from 100 ng to 1 µg) was treated to remove rRNA by using the QIAseq FastSelect –5S/16S/23S following the manufacturer’s instructions (Qiagen, Germany). The rRNA depletion yield was checked using a 2200 TapeStation (Agilent Technologies, USA). Then, a whole transcriptome library was generated using the TruSeq Stranded mRNA Sample preparation kit (Illumina, San Diego, USA). Samples were loaded into a NextSeq high output v2 kit (150 cycles) (Illumina, San Diego, USA) as indicated by the technical support guide. The obtained reads were filtered to remove low-quality reads (minimum mean quality 20, minimum length 150 bp) as well as any remaining ribosomal locus using the METAnnotatorX2 pipeline (68).

### Metabolomic analysis of GESM cultivation samples

#### Chemicals and reagents

Milli-Q water was prepared by a Milli-Q plus system from Millipore (Bedford, MA, USA). LC-MS grade acetonitrile (MeCN), methanol (MeOH), and formic acid were supplied by Scharlab SL (Barcelona, Spain).

#### Sample preparation

GESM cultivation samples were processed employing a published extraction protocol for the isolation of metabolome from microbial incubates, with minor modifications (39). Briefly, an aliquot of 500 µL of each supernatant obtained after centrifuging cheese cultivation in GESM was transferred into a 2 mL tube and centrifuged (7,000 × *g*, 5 min, 4°C). Two hundred microliters of the supernatant was treated with 800 µL of a 1:1 vol/vol mixture of MeOH/MeCN. After vortexing, samples were kept at −20°C for 60 min and then centrifuged (13,000 × *g*, 15 min, 4°C). Five hundred microliters of the supernatant was dried under a gentle nitrogen flux. Dried extracts were reconstituted in 200 µL of MeCN:water 1:1 vol/vol, further centrifuged (13,000 × *g*, 10 min, 4°C), and analyzed by LC-HRMS.

#### LC-HRMS metabolomic analysis

High-performance liquid chromatography (HPLC) separation of metabolites was carried out employing a Waters HSS T3 column (100 cm × 2.1 cm, 3.5 µm particle size; Waters, USA) on a Dionex HPLC system (Thermo Scientific, Waltham, MA, USA) equipped with a chilled autosampler (5°C) and heated column compartment (30°C). Chosen mobile phases were A: water containing 0.1% formic acid and B: MeCN containing 0.1% formic acid. The injection volume was 5 µL. The flow rate was 0.15 mL/min. Gradient was as follows: *t* = 0 min: 99%A:1%B, *t* = 3 min: 99%A:1%B; *t* = 20 min 5%A:95%B; *t* = 25 min 5%A:95%B; *t* = 26 min 99%A:1%B with a re-equilibration time of 4 min. Mass spectrometry detected analytes using an LTQ Orbitrap (Thermo Scientific, Waltham, USA), equipped with a heated electrospray ionization (H-ESI) ion source. Instrumental settings were as follows: H-ESI source voltage: 3 kV; capillary temperature: 275°C; sheath, auxiliary, and sweep gases: 40, 10, and 5 arbitrary units, respectively. Source voltage was 3 kV, and mass spectrometer operated in positive ion mode with full scan (m/z: 50–1,000) in data-dependent acquisition mode with a minimum signal intensity of 5,000 and a collision-induced dissociation (CID) energy = 35V. The analytical batch consisted of blank samples, QC samples, and unknown samples which were randomized before analysis. QC samples were prepared by mixing 10 µL aliquots of every sample in analysis. Ten QC sample injections were employed at the beginning of the analytical batch to ensure system stability, and a QC sample was injected every 10 samples.

#### Data processing

Thermo .raw data were imported into the untargeted analysis software Progenesis QI v.2.4 (Nonlinear Dynamics, UK) which performed alignment of batch runs and LC-HRMS features extraction by selecting the most suitable QC sample in the batch as alignment reference. LC-HRMS features (i.e., defined by a combination of LC retention time and accurate mass values) were filtered by accepting an intra-batch precision, expressed as percent coefficient of variation (CV%) among injected replicates of QC samples less than 25%. One thousand seven hundred eighty-seven LC-HRMS features showed a log_2_ difference >1 or −1 with respect to the incubation medium in at least one analyzed GESM cultivation (Table S6). Metabolite identity assignment was performed by Progenesis QI ID algorithm, carrying out elemental composition analysis with calculated mass, mass error (ppm), isotopic similarity (calculated isotopic pattern vs experimental one), and mass fragmentation by searching into Human Metabolome Database (https://www.hmdb.ca/), KEGG (www.genome.jp/kegg/compound), and ChEBI (www.ebi.ac.uk/chebi/).

### Consumed cheese and human fecal sample collection and DNA extraction

To evaluate whether bacterial species composing the cheese microbiota may survive the passage through the gastrointestinal tract and colonize/proliferate in the intestine ultimately influencing human gut microbiota, 13 stool samples belonging to healthy consumers of cheese were collected immediately after defecation by using a dedicated sterile tube provided with a sampling spoon. After collection, stool samples were immediately shipped to the laboratory under anaerobic and frozen conditions. Once in the laboratory, samples were stored at −20°C until they were further processed. DNA extraction from each fecal sample was performed using the QIAmp DNA Stool Mini Kit (Qiagen, Germany) following the manufacturer’s instructions. The 13 consumers declared that they consumed cheese daily or at least 5 days a week and the portion consumed was approximately of 5 or 6 g at a time. However, since the selected individuals were regular cheese consumers, it was not possible to collect a baseline fecal sample, i.e., a stool sample before cheese consumption. Indeed, fecal samples were collected in the framework of another study aimed at dissecting the impact of diet in a large cohort of individuals (71). Furthermore, fecal samples were collected in a tube containing bacteria-inactivating liquid, thus preventing the possibility to isolate bacterial species from these samples to verify the viability of those bacterial strains horizontally transmitted from cheese to consumers.

In addition, the three different raw milk-derived Italian PDO cheeses (Protected Designation of Origin) consumed by the 13 participants enrolled in the study were collected, i.e., CC1, CC2, and CC3. Specifically, five individuals consumed CC1, while CC2 and CC3 were consumed by four individuals. Upon their arrival in the laboratory, the three CC samples were cultivated in GESM for 16 h as above reported for the other 15 collected cheeses. Total DNA was extracted from the original three cheeses and from their corresponding GESM cultivation through the DNeasy PowerFood Microbial Kit (Qiagen, Germany) and QIAmp DNA Stool Mini Kit (Qiagen, Germany), as above described. Fecal and cheese samples as well as CC-related GESM cultivation were further processed for DNA sequencing as above reported.

### Reconstruction of bacterial sequences from CCs cultivated in GESM

Deep shotgun metagenomic data from the GESM cultivations of the three CCs were utilized to recover bacterial genomes, resulting in *de novo* metagenomic assembly and the taxonomic classification of 57 distinct bacterial genome sequences (Table S9). Specifically, raw data of shotgun metagenomic sequencing (fastq files) that passed quality filtering were used as input for SPAdes assembler v.3.12 (72), using default parameters and enabling the metagenomic flag option (-meta). SPAdes parameters were configured with minimum k-mer sizes of 21, 33, and 55, extending up to maximum sizes of 77, 99, and 127 based on the paired-end read length, as previously described (73). Following assembly, open reading frames (ORFs) of each assembled contig were predicted with Prodigal (74) with default parameters and then annotated using MEGAnnotator2 software (58).

### Strain tracking

Strain tracking was based on the use of short reads obtained from deep shotgun metagenomics data from the bacterial DNA extracted from fecal samples of 13 raw milk cheese consumers, aligning these reads on 57 MAGs obtained from CCs cultivated in GESM (Table S9). Preliminary strain tracking was performed using two different software packages, BBMAP (44) and BWA (45). Then, identification of all MAGs for which at least 10,000 bases were mapped and aligned was the outcome of the initial strain-tracking technique based on total coverage of MAP. Then, BBMAP and BWA (75) were used to track the 11 isolated bacterial strains in fecal sample-derived sequence data corresponding to 13 raw milk cheese consumers. Subsequently, these data were validated using a combined qPCR-PCR approach.

Strain-specific primers used for *Hafnia paralvei* T10 tracking with qPCR-PCR combined approach were designed through Primer3 (76) and Primer-BLAST (77) online tools. In detail, FW and RW primers were based on contig_21 of *Hafnia paralvei* T10 and reported a length of 20 bp and a GC% of 55%, with a self-3´ complementarity of 0, leading to a product of 144 bp. Primer-BLAST was used to test several primers targeting *Hafnia paralvei* T10 as well as to check for identical sequences in the “nr” database with Organism set to “Bacteria.” Then, the DNA of consumer samples was extracted and diluted at a concentration of 10 ng. The presence of *Hafnia paralvei* DNA was evaluated using qPCR with primer pair (5′-ACTCGTAGCCAGAAGCCAGA-3′ and 5′-CTCTCGCGGAAGCTTGTATC-3′).

In detail, qPCR was performed using the PowerUp SYBR Green Master mix (Thermo Fisher Scientific) on a CFX96 system (BioRad, CA, USA) following previously described protocols (78). qRT-PCR was performed by using the 2X PowerUp SYBR Green Master mix (Thermo Fisher Scientific). The qRT-PCR cycle consisted of a denaturation step at 95°C for 2 min, followed by separate annealing (5 s) and extension at 62°C for 2 min step. Fluorescence was monitored at the end of each extension step. Melting curve analysis was performed at the end of the amplification cycle. Data analysis was performed using the relative standard curve method.

### Bacterial strain isolation

GESM cultivations of the three CCs were subjected to a bacterial species isolation protocol. In detail, an aliquot of each GESM cultivation was serially diluted in PBS and plated by using the GESM culture medium. Agar plates were incubated under anaerobic (2.99% H_2_, 17.01% CO_2_, and 80% N_2_) atmosphere at 37°C for 96 h. After the incubation, colonies were randomly picked and the identification of each bacterial isolate was performed through the MALDI-TOF MS Biotyper Sirius (Bruker, USA). In detail, each bacterial colony grown on GESM agar plates was transferred onto a spot of the MicroScout Plate 96 target polished steel BC MALDI plate (Bruker, USA). Subsequently, the bacterial isolates were covered with 1 µL of matrix solution containing 10 mg/mL α-cyano-4-hydroxycinnamic acid (Merck, Germany) dissolved in an organic solvent (50% acetonitrile and 2.5% trifluoroacetic acid) and air dried (42). The MALDI target plate was then introduced into the Biotyper Sirius for automated measurement and data interpretation. The obtained mass spectra for each bacterial isolate were then processed through the MALDI Biotyper 3.0 software package (Bruker, USA) containing microbial species reference spectra. According to the criteria recommended by the manufacturer, a score ≥2 indicates a significant similarity between the generated spectrum and one of the spectra contained in the database. Each sample was analyzed in duplicate. The default parameters of the instrument were used for bacterial identification, i.e., positive linear mode, laser frequency 200 Hz, ion source 1 of 19.84 kV, ion source 2 of 18.07 kV, and a mass range between 2,000 and 20,000 Da. In addition, before sample loading, the bacterial test standard, including a protein extract of *Escherichia coli* DH5α, was loaded as a calibration test.

### Chromosomal DNA extraction and genome sequencing, assembly, and annotation

Bacterial isolates were revitalized from a glycerol stock and grown in GESM overnight. Subsequently, bacterial cells were harvested by centrifugation at 6,000 rpm for 8 min and subjected to chromosomal DNA extraction using the GenElute bacterial genomic DNA kit (Sigma-Aldrich, Germany) by following the manufacturer’s protocol. The chromosomal DNA of newly isolated bacterial species was then decoded through a MiSeq platform (Illumina Inc., San Diego, USA) according to the manufacturer’s instruction by using the Nextera XT DNA Library Prep Kit (Illumina), as previously described (79, 80). Libraries were quantified using a fluorometric Qubit quantification system (Life Technologies, USA) loaded on a 2200 Tape Station instrument (Agilent Technologies, USA), and normalized to 4 nM. Sequencing was performed using the Illumina MiSeq platform with a 600-cycle flow cell version 3 (Illumina Inc., USA). Raw DNA sequence reads (.fastq files) of paired-end reads generated from each genome sequencing were employed as input for genome assembly by using the MEGAnnotator2 pipeline (58). Briefly, SPAdes software was used for the *de novo* assembly of the genome sequences with the pipeline option “‐‐carefull” and a list of “21,33,55,77,99,127” k-mer sizes (81), whereas protein-encoding genes were predicted for contigs longer than 1,000 bp using Prodigal (74). Functional annotation of the predicted genes was achieved through DIAMOND (using the --sensitive option in search of query coverage >50 and e-value <1 × 10^−8^) (82) against the RefSeq database of NCBI resized with a CD-HIT sequence identity threshold of 70% (83), and further investigated through InterProScan (84). Furthermore, tRNA genes were determined using tRNAscan-SE 2.0 (85), while rRNA loci were identified with barrnap (https://github.com/tseemann/barrnap). Genome quality assessment was performed manually and through the use of CheckM (86) software for obtaining completeness and contamination score. Additionally, fastANI (87) software was employed for analyzing the average nucleotide identity between strains of the same species.

### *Hafnia paralvei* growth in GESM and RNA extraction and sequencing

*Hafnia paralvei* T10 was revived from a glycerol stock in GESM overnight. Subsequently, bacterial cells were enumerated through a Thoma cell counting chamber (Herka) and diluted in GESM or GESM supplemented with 2% of a heat-inactivated (autoclaved) cheese matrix to reach a final inoculum of 10^6^ cells/mL in 50 mL of growth medium. Cultures were then incubated under anaerobic conditions at 37°C. After 6 h of incubation, cells were harvested through centrifugation and subjected to RNA extraction and sequencing by using the same protocol above reported. The supernatant, instead, was stored at −80°C until metabolomic analysis.

### Metabolomic analysis of *Hafnia paralvei* cultures

Sample preparation of *Hafnia paralvei* cultures for the analysis of metabolites was carried out as previously described for GESM assay samples. A liquid chromatography–ion mobility spectrometry–quadrupole time-of-flight mass spectrometry (LC-IMS-qToF)-based instrumental platform was used to analyze reconstituted extracts. A Waters Acquity UPLC coupled to a VIon IMS-qToF mass spectrometer (Waters, Manchester, UK) equipped with an electrospray ionization (ESI) probe was employed to acquire high-resolution mass spectrometry (HRMS) data. The instrument operated in high-definition data-independent acquisition mass spectrometry (HDMSE) mode, which leverages ion mobility separation to enhance spectral clarity, allowing for improved compound identification and more accurate mass measurements. An Acquity HSS T3 UPLC column (2.1 × 100 mm; 1.7 µm particle size; Waters, Milford, USA), kept at 45°C, was used for gradient separation. Mobile phases were A: water and B: MeCN, both containing 0.1% vol/vol formic acid. Gradient was as follows: *t* = 0 min: 99%A:1%B, *t* = 1 min: 99%A:1%B; *t* = 15 min 5%A:95%B; *t* = 18 min 5%A:95%B; *t* = 18.5 min 99%A:1%B with a re-equilibration time of 1.5 min. Total run time was as follows: 20 min; flow rate: 0.4 mL/min; autosampler temperature: 5.0°C; injected volume: 5 µL. The capillary voltage was set to 1.0 kV and the cone voltage to 40 V. Source temperature was 120°C; the desolvation gas (N_2_) temperature was set at 550°C at a flow rate of 800 L/h; the source gas (N_2_) flow rate was 20 L/h. The mass range was 50–1,000 Da, with a scan time of 0.1 s. Lock mass (50 ng/mL leucine enkephalin) was calibrated to ensure mass accuracy throughout the analysis. Ten microliters of each sample type extract was combined to prepare QC samples, which were run before and during the analytical batch. The acquisition occurred in electrospray positive (ESI+), and the software UNIFI v.1.8.2 (Waters, Manchester, UK) was used for both system control and data acquisition.

#### Data processing

As previously described, UNIFI .uep data files were processed in Progenesis QI v.2.4. Only those ion features characterized by a CV% ≤25% in the QC samples and at least one putative ID by comparison with available databases (HMDB, KEGG, ChEBI) were selected; this resulted in 786 ion features. PLS-DA using SIMCA v.17 software (Sartorius Stedim Biotech, Sweden) was employed to determine the most discriminant metabolites in *H. paralvei* incubates in the presence or absence of heat-inactivated cheese matrix concerning corresponding media. Before analysis, data were log_10_ transformed, centered, and Pareto-scaled prior to modeling. Variable importance in projection values >2 were employed as cut-off values. Average log_2_ intensity values and SD were reported for each metabolite signal in each condition (*n* = 3). Student’s *t*-test was employed to search for statistical significance (*P* < 0.05).

### Statistical analysis

ORIGIN 2023 (https://www.originlab.com/2023), IBM SPSS statistics software (version 25) (www.ibm.com/software/it/analytics/spss/), and R-Studios were used to compute statistical analyses.

For taxonomical, functional, and metabolic data, input data were pre-processed and transformed in a Bray-Curtis dissimilarity matrix with vegdist function (from vegan_2.5-7).

PERMANOVA and ANOSIM analyses were performed on R-Studios using 999 permutations to assess *P*‐values for population differences in PCoA with adonis2 package (from vegan_2.5-7). A non-parametric Kruskal-Wallis test was performed on SPSS software to evaluate the significance of the difference between enzyme expression (RNAseq data) and metabolite abundances across clusters. To assess the robustness of our findings despite unbalanced group sizes, we computed the median differences for each metabolite and applied boostrap resampling (1,000 iterations) to generate 95% confidence intervals.

Spearman correlation was performed with the rcorr function (from Hmisc_4.6-0), and only statistically significant results were retained. The eigenvalues were retrieved from the Bray-Curtis dissimilarity matrix using the prcomp function (from base package stats) and the get_pca function (from factoextra_1.0.7).

When FDR correction was applied, the Benjamini-Hochberg or Bonferroni approach was used on R-Studios through p.adjust function (from base package stats).

## Data Availability

The data sets supporting the conclusions of this article are available in the NCBI repository under accession no. PRJNA865096 (15 raw milk cheese samples), PRJNA1047028 (shotgun samples of GESM assay, RNAseq samples of GESM assay and human consumer fecal shotgun samples), and PRJNA1047050 (isolated bacterial strains).

## SUPPLEMENTAL MATERIAL

Supplemental figures aem.00180-25-s0001.pdf Figures S1 to S3.

Supplemental text aem.00180-25-s0002.pdf Additional experimental details.

Supplemental tables aem.00180-25-s0003.xlsx Tables S1 to S14.

ASM does not own the copyrights to Supplemental Material that may be linked to, or accessed through, an article. The authors have granted ASM a non-exclusive, world-wide license to publish the Supplemental Material files. Please contact the corresponding author directly for reuse.
