# Supplementary material for: Functional modulation of the human gut microbiome by bacteria vehicled by cheese
Source: Appl Environ Microbiol. 2025 Feb 28;91(3):e00180-25. doi: 10.1128/aem.00180-25 (PMC11921328; doi:10.1128/aem.00180-25)
Supplement: Supplemental figures — Figures S1 to S3. [file aem.00180-25-s0001.pdf]

a)

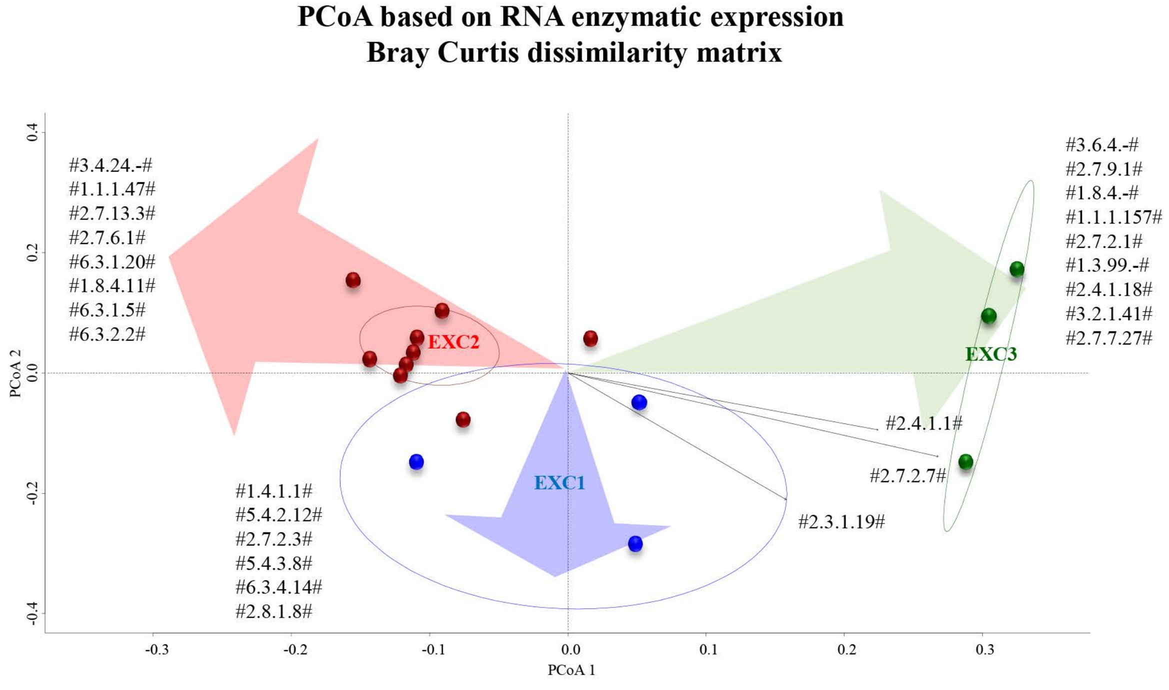

Centroids:

	Axis.1	Axis.2
EXC1	-0.0031	-0.1607
EXC2	-0.1010	0.0406
EXC3	0.3060	0.0388

Goodness of fit:

	R^2	Pr(>r)
Cluster	0.7531	0.001 ***

Permutation: free
Number of permutations: 999

ANOSIM:

	R	Significance
Cluster	0.7838	0.001 ***

Permutation: free
Number of permutations: 999

PERMANOVA:

	Df	SumOfSqs	R^2	F	Pr(>F)
Cluster	2	0.50449	0.40458	4.0768	0.001 ***
Residual	12	0.74247	0.59542		
Total	14	1.24696	1.00000		

Permutation: free
Number of permutations: 999

b)

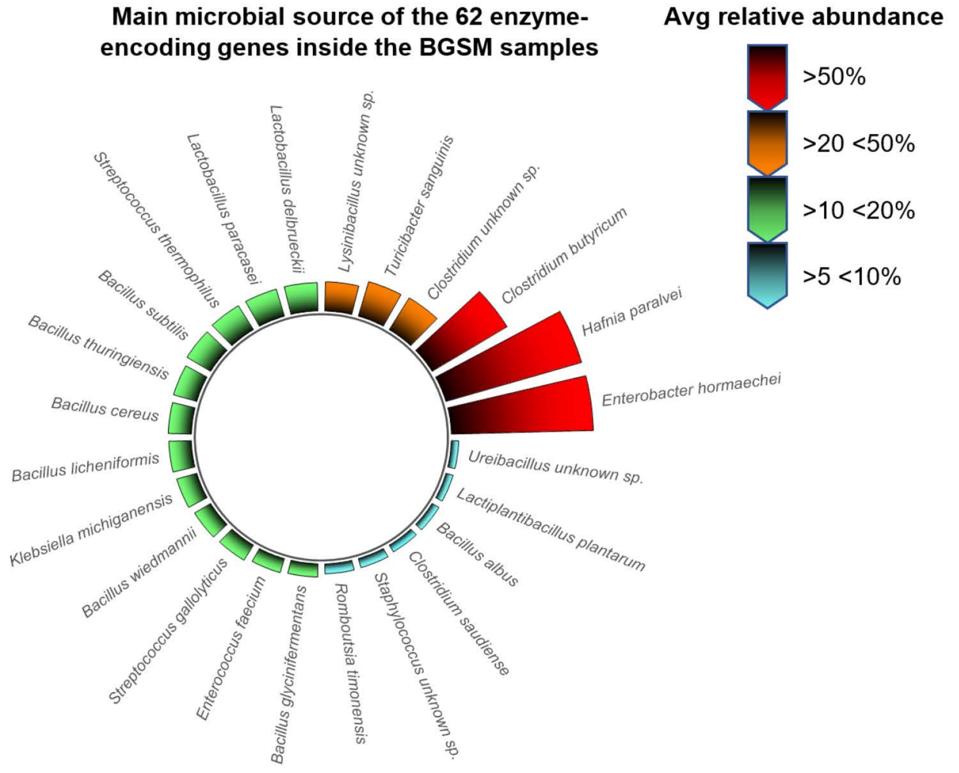

Figure S1: Panel a shows a 3D PCoA based on the expression levels of genes related to EC Numbers (enzymes), in which a fitting analysis was carried out in order to identify only those EC Numbers that explain most of the expression variability present among the cheese samples following the GESM assay. Panel b displays a circular bar plot representing the microbial species with a relative abundance of more than 5% of the average DNA derived from the 62 tracked product/enzyme encoding genes in each sample. Species with higher average relative abundance values (red) possess the most significant genetic potential to express all 62 product/enzymatic-encoding genes.

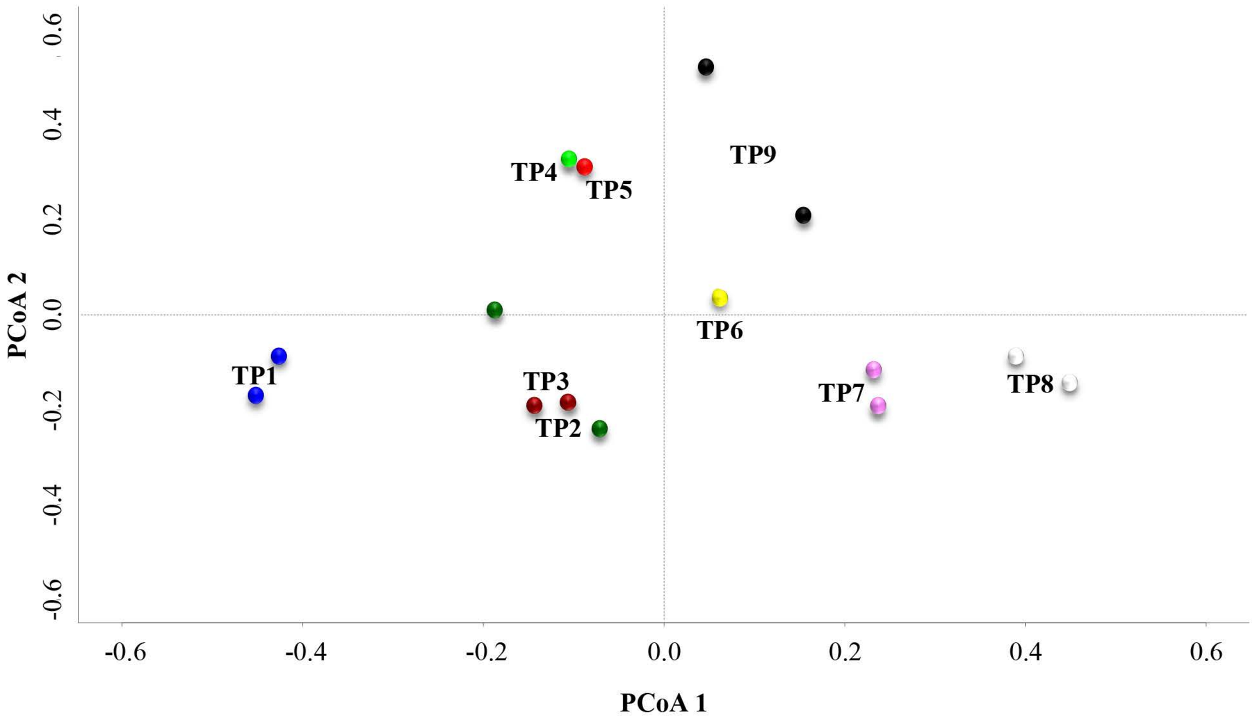

**Goodness of fit**:	R^2	FDR – p-value
Clusters (TP)	0.9404	0.001 ***
**ANOSIM**	R^2	FDR – p-value
Clusters (TP)	0.963	0.001 ***
**PERMANOVA**	R^2	FDR – p-value
Clusters (TP)	0.79695	0.001 ***

PCoA related statistic

Figure S2: Metabolomic analysis of H. paralvei T10 after growth in GESM or GESM supplemented with heat-treated cheese. Panels a, b, and c report the results of PCA and PLS-DA analysis on putatively identified compounds by UPLC-IMS-QToF. The analysis was performed on four experimental conditions: GESM, GESM supplemented with heat-treated cheese, H. paralvei T10 in GESM, and H. paralvei T10 in GESM supplemented with heat-treated cheese. Panels a, b, and c depict the PCA obtained by considering all conditions.

a)

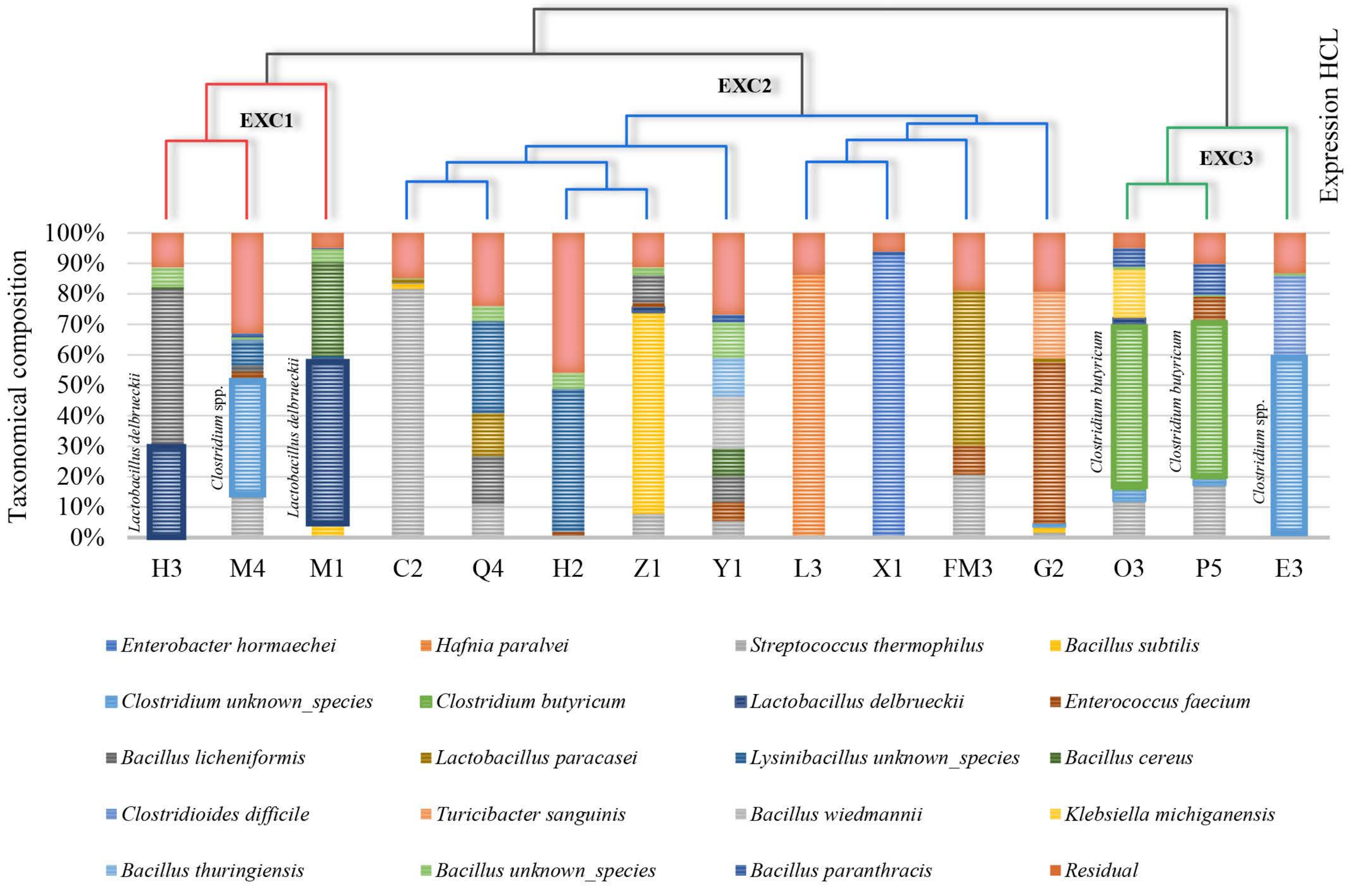

b)

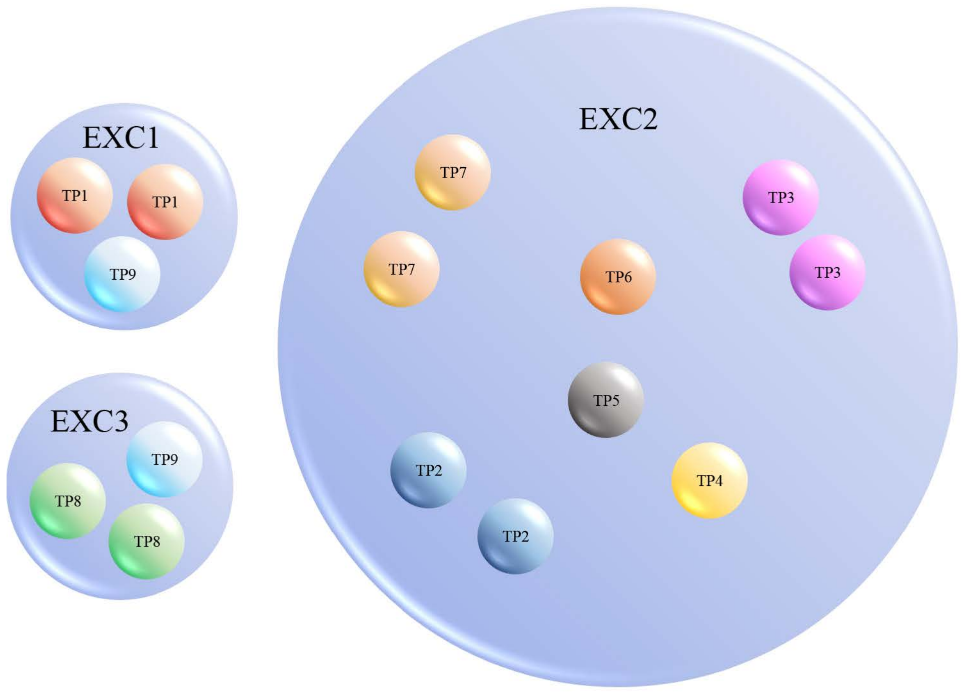

Figure S3: Taxonomic profiles after cheese growth in GESM. Panel a depicts a bar-plot graph reporting the average relative abundance of the most abundant bacterial species detected in cheese GESM cultivation. Samples were reordered according to the Hierarchical Clustering Analysis based on the enzymatic expression patterns of each sample. Panel b shows the relationship between Enzymatic Expression Clusters (EXCs) e Taxonomical Profiles (TPs).
