# Supplementary material for: Functional modulation of the human gut microbiome by bacteria vehicled by cheese
Source: Appl Environ Microbiol. 2025 Feb 28;91(3):e00180-25. doi: 10.1128/aem.00180-25 (PMC11921328; doi:10.1128/aem.00180-25)
Supplement: Supplemental text — Additional experimental details. [file aem.00180-25-s0002.pdf]

Functional modulation of the human gut microbiome by bacteria vehicled by cheese

Keywords: Microbiota, food, metagenomics, metatranscriptomics, metabolomics, human diet.

Running title: Cheese-associated microbes and the human gut microbiome

Christian Milani[1,3*°] Giulia Longhi[1°], Giulia Alessandri[1], Federico Fontana[1,2], Martina Viglioli[6], Chiara Tarracchini[1], Leonardo Mancabelli[3,4], Gabriele Andrea Lugli[1,4], Silvia Petraro[1], Chiara Argentini[1], Rosaria Anzalone[2], Alice Viappiani[2], Elisa Carli[1], Federica Vacondio[6], Douwe van Sinderen[5], Francesca Turroni[1,3], Marco Mor[3,6] and Marco Ventura[1,3*]

Laboratory of Probiogenomics, Department of Chemistry, Life Sciences, and Environmental Sustainability, University of Parma, Parma, Italy[1]; GenProbio Srl, Parma, Italy[2]; Microbiome Research Hub, University of Parma, Parma, Italy[3]; Department of Medicine and Surgery, University of Parma, Parma, Italy[4]; APC Microbiome Institute and School of Microbiology, Bioscience Institute, National University of Ireland, Cork, Ireland[5], Department of Food and Drug, University of Parma, Parma, Italy [6]

°These authors contributed equally.

*These authors contributed equally.

Correspondence. Mailing address for Christian Milani Laboratory of Probiogenomics, Department of Chemistry, Life Sciences, and Environmental Sustainability, University of Parma, Parco Area delle Scienze 11a, 43124 Parma, Italy. Phone: ++39-521-904785. E-mail: christian.milani@unipr.it

Mailing address for Marco Ventura Laboratory of Probiogenomics, Department of Chemistry, Life Sciences, and Environmental Sustainability, University of Parma, Parco Area delle Scienze 11a,

43124 Parma, Italy. Phone: ++39-521-905666. Fax: ++39-521-905604. E-mail: marco.ventura@unipr.it

Supplementary Text

Investigation of the microbial community resulting from *in batch* growth of cheeses in a medium designed to mimic the human gut environment.

Overall, the average relative abundance of *Streptococcus thermophilus* dropped from 29.71% to 11.43%, that of *Lactobacillus helveticus* from 16.48% to 1.11%, while a reduction from an average relative abundance of 12.76% to 0.10% was recorded for *Lactococcus lactis* (Table S2 and Table S3). Intriguingly, while the relative abundances of these bacterial species decreased in GESM-cultured cheeses, other species assumed dominant percentages, including *Hafnia paralvei* (average relative abundance of 86.07%), *Enterobacter hormaechei* (92.73%), and *Bacillus subtilis* (66.08%) in GESM cultivation of L3, X1, and Z1 cheeses, respectively (Table S2 and Table S3). A trend that was also confirmed by analyzing the bacterial species prevalence. Indeed, while the most widespread species (prevalence > 50%) in cheese samples were represented by *Streptococcus thermophilus*, *Streptococcus* spp., *Lactobacillus* spp., *Lactobacillus paracasei*, *Lactobacillus delbrueckii*, *Lactobacillus helveticus*, and *Lactobacillus rhamnosus*, after cheese cultivation in GESM, only *Streptococcus thermophilus* maintained a high prevalence (> 50%). Indeed, beyond the latter species, GESM cultivation were characterized by *Enterococcus faecium* and *Bacillus* spp. (prevalence > 50%) as well as by *Clostridium* spp., *Bacillus licheniformis*, *Enterococcus durans*, *Enterococcus* spp., and *Bacillus paranthracis* with a prevalence of > 30% (Supplementary excel data 1). Notably, these species were not detected in the raw cheese microbiota, probably due to an average relative abundance below the limit of detection set for background noise exclusion (< 0.01%), except for *Streptococcus thermophilus*, which was identified in all the 15 cheese-representative samples (100% prevalence) with an average relative abundance of 29.7% (Table S2 and Table S3).

In depth-insight into the clusters obtained after cheese GESM cultivation revealed that TP1, TP3, TP4, TP5, TP6, TP8, and TP9 were dominated by a single species, i.e., *Lactobacillus delbrueckii*, *Lysinibacillus* spp., *Hafnia parlvei*, *Enterobacter hormaechei*, *Enterococcus faecium*, *Clostridium*

butyricum, and *Clostridium* spp., respectively, while TP2, and TP7 were both characterized by two different prominent species. Indeed, TP2 resulted to be dominated by *Bacillus subtilis* and other still undefined *Bacillus* species, while TP7 showed a predominance of *Streptococcus thermophilus* and *Lactobacillus paracasei* (Figure S2).

By linking the gene expression-derived clusters to the nine taxonomic profiles (TPs) obtained from cheeses after the GESM, we found that EXC2 accounted for the majority of the heterogeneity in taxonomic composition, including six of the nine taxonomic profiles (Figure S3).

Instead, only three taxonomic profiles, TP1, TP8, and TP9, revealed different enzymatic-coding gene expressions, clustering in EXC1 and EXC3, with TP9 as a link between the latter two EXCs (Figure S3).

Correlation between EXCs and metabolites of the gut-simulating medium following GESM assay.

After observing the presence of three different clusters of enzyme-related expression, we tried to assess their overall metabolic-related activity after 16h of GESM assay. Thus, the variation in previously identified metabolites, expressed as the fold change distribution, has been correlated with the different EXCs. Therefore, this analysis aims to observe how the functional (enzymatic) expression related to EXCs can affect the metabolic component in the consumer's gut, a condition simulated by the GESM assay.

In detail, 90 metabolites were found to differ significantly in fold change (Vs. the medium) between the three EXCs (Kruskall-Wallis FDR-Bonferroni p-value < 0.05) (Table S7).

These findings demonstrated that fermented products, in this case, cheeses, are not only "functional foods" capable of supplying vitamins and compounds of high biological value to the diet, but the microbial composition resulting from their consumption can functionally impact the metabolomics profiles at the intestinal level, as simulated by the GESM assay.
